# Reconsidering reproductive patterns in a model dissociated species, the red-sided garter snake: Sex-specific and seasonal changes in gonadal steroidogenic gene expression

**DOI:** 10.3389/fendo.2023.1135535

**Published:** 2023-03-13

**Authors:** Julianna M. Lincoln, Megan L. Barlowe, Holly R. Rucker, M. Rockwell Parker

**Affiliations:** ^1^ Department of Biology, Harrisonburg, James Madison University, VA, United States; ^2^ Department of Cellular and Molecular Biology, University of Wisconsin, Madison, WI, United States

**Keywords:** gene expression, steroidogeneis, reproductive pattern, garter snake, testis, ovary, season

## Abstract

Sex steroid hormones are powerful regulators of reproductive behavior and physiology in vertebrates, and steroidogenesis has distinct sex- and season-specific patterns ultimately dictated by the expression of key enzymes. Most comparative endocrinology studies, however, focus only on circulating levels of sex steroids to determine their temporal association with life-history events in what are termed associated reproductive patterns. The red-sided garter snake (*Thamnophis sirtalis parietalis*) is a notable exception; this species exhibits maximal sex behavior decoupled from maximal sex steroid production and gametogenesis in what is termed a dissociated reproductive pattern. And while this is true for male red-sided garter snakes and their production of testosterone, females have maximal estradiol production during peak breeding (spring) but only immediately after mating. Here, we demonstrate that expression of ovarian aromatase (conversion of androgens to estrogens) matches the established seasonal hormone pattern in females. Additionally, steroidogenic gene expression in the ovary is broadly reduced if not suppressed compared to the testis throughout the active year. Bizarrely, male red-sided garter snakes demonstrate an unexplained pattern of steroidogenic gene expression in the testis. StAR (import of cholesterol to steroidogenesis) is maximally expressed in spring, yet Hsd17b3 expression (conversion of androstenedione to testosterone) is highest in summer, with the latter matching the established summer peak in male testosterone. The function of elevated StAR in spring is unknown, but our results suggest a decoupling between maximal StAR expression and testosterone biosynthesis (Hsd17b3 expression). We also purport that the reproductive pattern binary should be reassessed given its lack of fit for many vertebrate species that demonstrate seasonal, mixed patterns of (a)synchrony between circulating sex hormones and reproductive behavior.

## 1 Introduction

**Figure 1:**
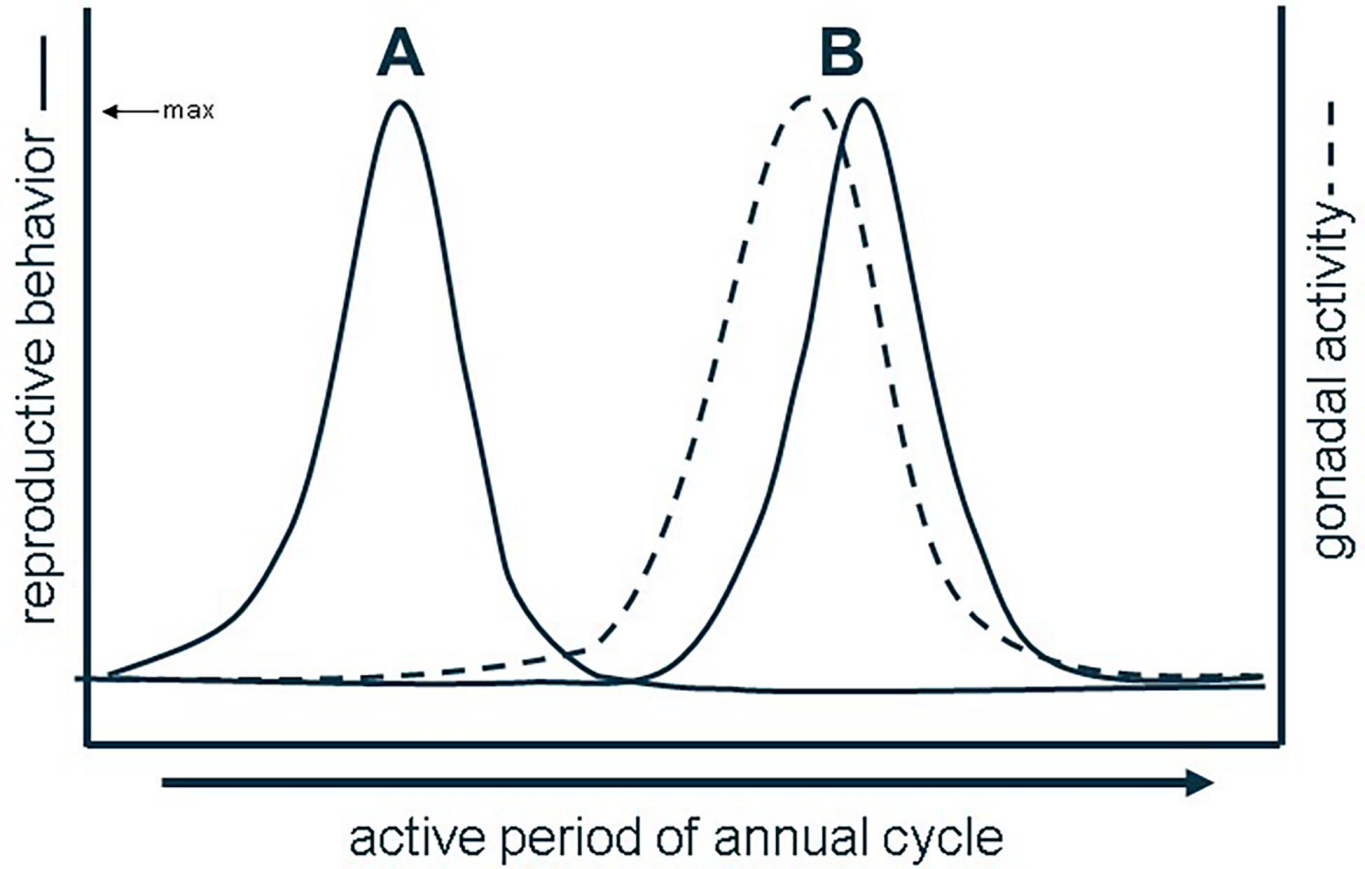
Dissociated **(A)** vs. associated reproductive patterns **(B)** based on the (a) synchrony of behavior with gonadal activity. Redrawn from (9).

In vertebrates, both reproductive behavior and physiology are predominantly controlled by sex steroid hormones (1). Sex steroid hormones are primarily synthesized in the gonads from cholesterol then refined by key cytochrome P450 enzymes into androgens and/or estrogens to exert their effects in target tissues (2). Sex hormone signaling has manifold effects across reproductive functions in vertebrates, including gametogenesis, vitellogenesis, growth and development, and mating behavior (e.g., 3–7). In circulation, sex steroid levels fluctuate seasonally and often coinciding with major reproductive events in species with distinct breeding seasons (e.g., 8). The relationship between sexual behavior, circulating sex steroid hormones, and gametogenesis is often described as one of three types of reproductive patterns. Most vertebrate species display an associated reproductive pattern, wherein mating behavior, maximal gametogenesis, and peak circulating sex steroid concentrations coincide temporally (9). Contrastingly, species with dissociated reproductive patterns exhibit mating behavior independent of or out of sync with maximal gametogenesis and sex steroid secretion (9) (**Figure 1**). Many vertebrates, however, do not fit strictly into either of these most common categories, especially species with more than one breeding season where both associated and dissociated patterns are observable in a given year (10). Third is the opportunistic pattern where gametogenesis and mating are either constitutive or paused until a mating opportunity is present, a pattern described in many vertebrates living in unpredictable environments (11–13). To determine the strength of the temporal association between reproductive behavior, gametogenesis, and sex steroid production, the majority of studies focus only on either circulating levels of sex steroids or gametogenesis and compare that to documented instances of mating in a given species, and, worse, most studies typically assume an associated reproductive pattern.

The red-sided garter snake (*Thamnophis sirtalis parietalis*) is a species that provides a unique opportunity to examine reproductive physiology as it is one of the most extensively studied non-model, seasonally breeding vertebrates with a dissociated reproductive pattern (14–16). After an 8-month winter dormancy in the northern extent of their range, red-sided garter snakes emerge in the tens of thousands from limestone hibernacula in spring, and males immediately begin displaying intense courtship behavior toward singly-emerging females across the 4-week breeding season (17). Though male courtship behavior is maximal in spring, the testes are in their most regressed state, circulating testosterone is either at or declining toward baseline, and neither castration nor supplemental testosterone influence courtship at this time (14, 18). Courtship behavior is extinguished by the end of spring mating, and several months later the testes eventually recrudesce and circulating testosterone peaks in late summer (15, 19). Similar to males, females are maximally receptive upon emergence in spring, yet the ovaries are typically regressed (20–22). However, unlike males, female garter snakes have significant variation in both receptivity and ovarian state in spring (23). Immediately post-mating, females produce a significant, short surge in circulating estradiol (E_2_) from the ovaries and eventually undergo ovarian recrudescence, summer gestation, and parturition (23). Mating is not a requirement for ovarian recrudescence and parturition, though: females who do not mate in a given spring can still become gravid and give birth that year (22, 24). Further, E_2_ is crucial for regulating female reproduction in this species, such as vitellogenesis, receptivity, and attractivity/pheromone production (21, 24–26). During the late summer, prior to dispersal back to the den, gametogenesis occurs in both sexes in preparation for the next spring (9, 15, 17). Therefore, the red-sided garter snake is a textbook example of the dissociated reproductive strategy, making this species valuable for understanding cyclical shifts in gene expression underlying broad physiological phenomena (27).

Dissociated reproduction in *T. s. parietalis* is considered an adaptative mechanism that resulted from the environmental pressures of living at extreme latitudes (9, 28). The chief limitation in such environments is temporal: the amount of time these ectotherms can be safely active during the year is approximately six months at most (May to October). Worse, weather events during the active period of the year can be random and severe, such as a mass mortality event one spring caused by a sudden snowstorm and freeze (29). The frequency and intensity of extreme climatic events are likely to increase due to climate change, increasing the need to understand the range and limitations of phenotypic plasticity (30, 31). In the red-sided garter snake, selective pressures shaping reproduction under such stochastic conditions with narrow mating windows likely favored seasonal modulation of gene expression in steroidogenesis (32). The enzymes responsible for regulating sex steroid production are sensitive to many external modulators that regulate their expression and activity and, thus, downstream hormonal products, especially in seasonally breeding vertebrates (e.g., 33). Indeed, seasonal variation in expression or activity of steroidogenic enzymes has been observed across mammals, birds, fish, and reptiles (e.g., 33–37), including some studies highlighting mismatches between enzyme expression and circulating sex steroids (e.g., 38, 39). Because of the relationship that seasonal stimuli have with gonadal activity, environmental modulation of endocrine systems has been posited as a major generator of adaptive phenotypic plasticity, especially in species living in extreme conditions (e.g., 40). Therefore, measurements of seasonal changes in gene expression, not just the circulating output of said genes (e.g., sex steroids), can inform predictions about the physiological substrates shaped by environmental selection.

**Figure 2:**
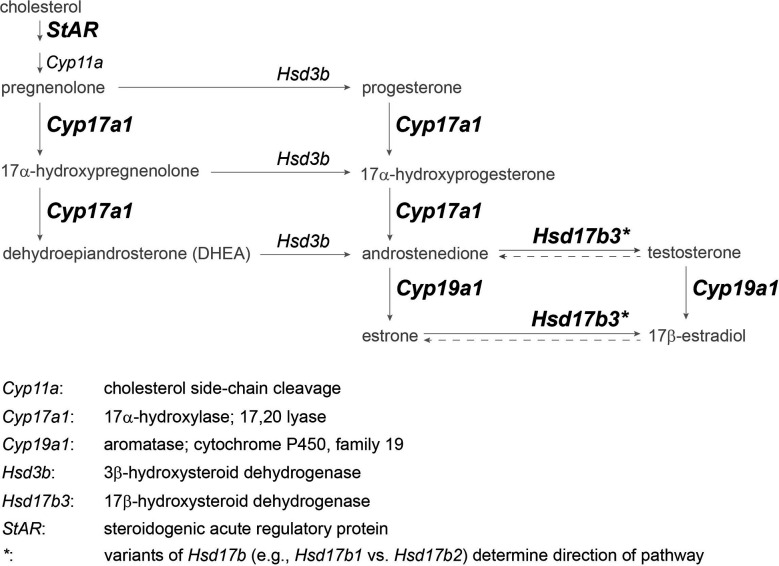
Simplified vertebrate steroidogenesis pathway focused on principle sex steroids (modified from (41)). Steroid hormones and precursors are in gray, enzyme gene names are in italics, and enzymes in bold indicate target genes for which primers could be designed. Genes are defined below pathway.

In this study, we aimed to determine whether mRNA expression of four key steroidogenic enzymes varies seasonally in accordance with established behavioral and hormonal patterns in both sexes of the red-sided garter snake. The four principal enzymes of interest are StAR, Cyp17a1, Hsd17b3, and Cyp19a1 (aromatase) as they comprehensively span the length of the step-wise steroidogenic pathway and cover four major steroid products therein (**Figure 2**), and these enzymes have also been examined in another excellent reptile model for comparative reproductive endocrinology, the green anole (*Anolis carolinensis*) (35). mRNA expression was quantified *via* qPCR using cDNA synthesized from both male and female gonadal tissue in the red-sided garter snake across three sequential phases during the active period of the same year. To date, the only steroidogenic enzyme studied in this species is aromatase, and even then, only the sexual and seasonal variation in protein within specific nuclei of the brain related to sex behavior (42). Therefore, this study offers insight at the molecular level on the seasonal steroidogenic capacity of the gonads in a model reptile with dynamic reproduction and life history.

## 2 Methods

### 2.1 Animal husbandry and seasonal conditions

A total of n=60 red-sided garter snakes (n=30 females, n=30 males) were collected from the wild at a known hibernaculum in Inwood, Manitoba, Canada (N 50° 30’ 25.5416”, W 97° 29’ 49.7003”, 277 m). The snakes were collected in May 2018 during the peak of the spring breeding season. All females were newly emerged from the hibernaculum (body temperatures ~2-4°C), were extremely attractive to males (mated females become unattractive to males) and had no mating plug or remnants of a plug indicating they had not mated that spring. Females were further determined to be attractive based on whether they received repeated, daily courtship from sexually active, wild males in the den. All males were sexually active and vigorously courting females in the den at the time of capture.

Snakes (n=10 females, n=10 males) were euthanized for tissue collection at three different times: spring (May), summer (July), and fall (October). Spring snakes were euthanized at the field station after being separated by sex and held in outdoor arenas (e.g., 19) at ambient conditions for four days and provided water *ad libidum*. The remaining snakes were brought to the laboratory at James Madison University, Harrisonburg, Virginia, and housed in 37.8 L glass aquariums (n=3 per tank; sexes separated) equipped with basking lamps, shedding rocks, water bowls, hide objects, and shredded cardboard bedding (e.g., 43–45). All were maintained within an environmental chamber programmed to simulate the summer conditions of the Interlake region in Manitoba (16 h:8 h L:D; 25:15°C). The chamber was kept at these summer conditions from June through August, after which the program was transitioned to simulate fall field conditions (11 h:13 h L:D; 15:8°C) from September until October. The housing, temperature, and photoperiod conditions were established in previous studies on red-sided garter snakes (43, 44), and these seasonal conditions are sufficient to cause shifts in gene expression in *T. s. parietalis* (45) and induce natural steroid hormone cycles that are established regulators of gametogenesis, sex behavior, and reproduction in this species (e.g., 14, 15, 44). Snakes were fed a mixed diet of earthworms and brook trout once per week throughout the summer, and water was provided *ad libitum*. Housing densities (n=3 for males; n=2 for females) enabled sufficient animal care to ensure all individuals were eating each week in compliance with animal care requirements, and snakes were also weighed and measured once per month to track condition. As part of the transition from summer to fall, the snakes were not fed the month prior to fall tissue collection (45); in the wild, red-sided garter snakes become aphagic by fall prior to winter dormancy (19). All procedures involving the use of vertebrate animals were approved by Manitoba Conservation and the IACUC of James Madison University.

### 2.2 Tissue collection

After euthanization, gonads (along with many other tissues) were collected at the three timepoints (spring, summer, fall) following protocols in Ashton et al. (45). Summer samples were collected one month after snakes had acclimated to simulated summer field conditions, and fall samples were collected one month after snakes had acclimated to fall conditions. All snakes were euthanized with an overdose of brevital sodium (10 mg/kg) by intraperitoneal injection followed by decapitation. Tissues were then excised, placed in cryotubes, snap frozen in liquid nitrogen, and stored at -80°C until use in RNA extraction.

### 2.3 Primer design, RNA extraction, and cDNA synthesis

**Figure 3:**
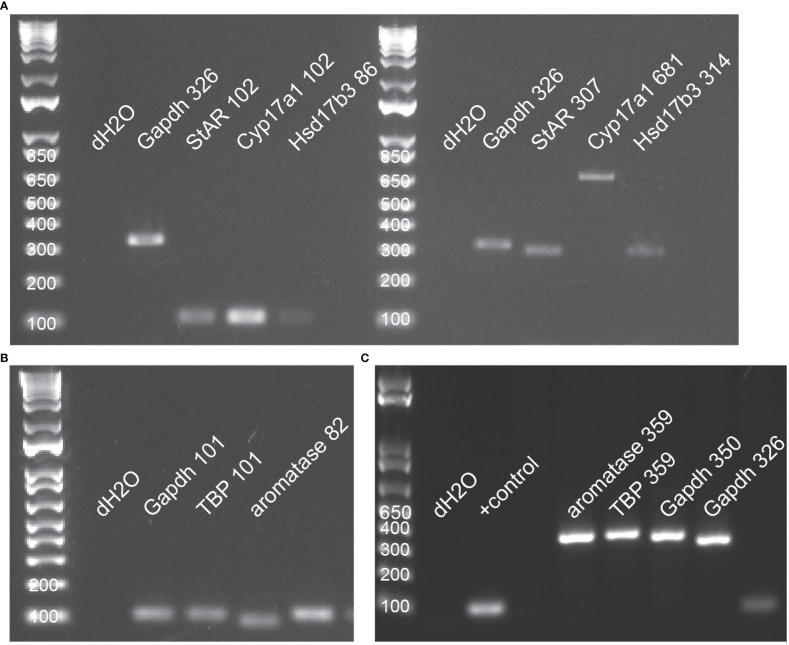
All primers were validated in traditional PCR using tissue-specific cDNAs from red-sided garter snakes. **(A)** Primer validation for StAR (steroidogenic acute regulatory protein), Cyp17a1 (17α-hydroxylase), and Hsd17b3 (17β-hydroxysteroid dehydrogenase); small amplicons (left) and large amplicons (right); pooled testis cDNA. **(B)** Primer validation for control genes (Gapdh, TATA box binding protein) and aromatase (Cyp19a1); small amplicons; pooled ovarian cDNA. **(C)** Primer valdiation for control genes and aromatase; large amplicons; pooled female liver cDNA. In all images, numbers following gene name indicate PCR product length (base pairs).

Methods for all molecular work followed Ashton et al. (45), with modification to the RNA extraction and cDNA synthesis protocols. Primers for all control and target genes were designed in NCBI BLASTR. The target genes were: steroidogenic acute regulatory protein (StAR), P450 17α-hydroxylase/C17-20lyase (Cyp17a1), 17 beta-hydroxysteroid dehydrogenase type 3 (Hsd17b3), and cytochrome P450 19A1 (aromatase/Cyp19a1). Control genes were glyceraldehyde-3-phosphate dehydrogenase (Gapdh) and TATA box binding protein (TBP). See **Table 1** for further details about each primer set. Prior to designing primers, protein sequences for each target gene were accessed across ten species, including the red-sided garter snake, and then aligned in Clustal Omega to determine regions of homology where designed primers in *Thamnophis sirtalis* had highest probability of authentically amplifying the target gene. Visualization of alignments also helped predict intron-exon boundaries for quantitative PCR primer design. All primer sets were validated using traditional PCR and gel electrophoresis before performing quantitative PCR (**Figure 3**).

**Table 1:** Sequence information for primer pairs used in qPCR for four target genes (StAR, Cyp17a1, Hsd17b3, and aromatase [Cyp19a1]) and two control genes shaded in gray (Gapdh [glyceraldehyde-3-phosphate dehydrogenase], TBP [TATA box binding protein]).

Gene	Size (bp)	Forward Primer	T_m_ (°C)	Reverse Primer	T_m_ (°C)	NCBI RefSeq
StAR	102	TGGTCCCACATGCATGATTCT	59.7	GTCTTTGGAAGCCATCCCTTT	58.5	XM_014064705.1
Cyp17a1	102	CCAAATAAAGATTTGGCTTTGCTGA	59.1	AGAATCACTGCTGAACATTTCTTTA	57.3	XM_014056702.1
Hsd17b3	86	ACACTCTATTCTGCATCTAAGGC	62.4	GGGAGTCACTGCCTGTATCA	60.0	XM_014051630.1
Cyp19a1	82	TGCGATGATAGCCATCTGTGTC	60.5	GACATGTTCCAACTCGGTTGTC	59.7	XM_014058523.1
Gapdh	101	TGACTCTACTCATGGCCGTTTC	60.0	CAGGATCACGCTCTTGGAAAAC	59.8	XM_014067593.1
TBP	101	TTAACAGGTGCAAAAGTCAGAGG	59.1	AACACATGGAACTGTTACGTCG	59.2	XM_014053067.1

Amplicon size (base pairs; bp), melting temperature (T_m_), and NCBI Reference Sequence identifiers are provided.

To prepare tissues for RNA extraction, single, frozen, whole gonads were weighed then homogenized in liquid nitrogen using clean mortar and pestles (Cryo-Cup). Homogenized tissues were kept frozen (-80°C) until use in RNA extraction. All gonadal tissues were weighed prior to RNA extraction, however, only whole, anterior testis wet weight could be accurately reported; the entire string of ovarian follicles was not collected per female. Wet mass of the anterior testis (mean ± s.d.) for each season was: 65.2 ± 22.6 mg (spring), 96.6 ± 37.3 mg (summer), and 104.2 ± 43.5 mg (fall). Further, sperm were not visible in the ductus deferens in summer males but were visible in the fall males, indicating that spermiation had occurred by fall tissue collection (R. Parker, pers. obs.). An attempt was made to thaw and individually isolate follicles to determine mass, but follicles ruptured upon thaw and the smallest could not be individually isolated (~1mm diameter). Follicles were widely variable in size within and among individuals, but fall follicles were flaccid, visibly regressed, and filled with transparent fluid as is typical for wild females in fall in this population (e.g., 46). Spring and summer follicles were turgid and contained opaque fluid indicative of vitellogenin and other yolk-type proteins but were not fully vitellogenic (e.g., follicle diameter > 1 cm; 22).

RNA was extracted using Quick-RNA Miniprep Plus kits (Zymo) and followed the manufacturer protocol. RNA quality was assessed using a Gen5 Microplate Reader; absorbance ratio (260nm/280nm) and concentration (ng/μL) were recorded, and concentration was used to standardize input RNA concentrations to ensure parallel cDNA synthesis. Absorbance ratios averaged 2.035 (0.056 s.d.).

Prior to cDNA synthesis, in-tube DNase I treatment (Invitrogen) was used to digest genomic DNA following the manufacturer protocol. cDNA was synthesized using the RevertAid First Strand kit (ThermoScientific) following the manufacturer protocol. A standard amount of sample RNA (1μg) was used in all cDNA syntheses and stored at -20 °C until further use. cDNAs were validated using traditional PCR with primers for Gapdh.

### 2.4 Quantitative PCR

Primers were tested to confirm primer efficiencies using control tissue (pooled summer ovarian cDNA) before progressing to testing of experimental tissues. Experimental primer efficiencies were 110.3% for StAR, 96.7% for Cyp17a1, 110.6% for Hsd17b3, and 108.1% for Cyp19a1 (**Figure S1**). All primer sets resulted in single products from melt curves. Methods followed those of Ashton et al. (2018). cDNAs were diluted to a 1:10 ratio for all qPCR reactions, and reactions were conducted following the SYBR Green protocol (ThermoFisher). Each sample was run in triplicate on 96-well plates in a CFX384 Touch Real-Time PCR System (Bio-Rad). The thermal cycles were as follows: activation at 95°C for 30 s, 45 cycles at 95°C (5 s each), and 60°C for 30 s followed by 31 s at 65°C. Melt curve analysis followed the completion of each run (65°C to 95°C in 0.5°C increments for 5 s). Samples across all three seasons (spring, summer, and fall) and each sex were included on every 96-well plate to control for interassay variation. Two target genes (StAR and aromatase; Cyp17a1 and Hsd17b3) and both control genes (Gapdh and TBP) were run per plate. All plates also included an internal control (pooled [n=6] summer ovarian cDNA).

### 2.5 Data analysis and visualization

For determining the effect of season on gene expression, one-way ANOVAs were conducted for each gene followed by *post-hoc* comparisons (Tukey tests). For determining the effect of sex and season on gene expression, two-way ANOVAs were conducted for each gene to determine any interactions prior to sex-within-season pairwise comparisons (Tukey tests). Correlations in gene expression were first targeted from a Pearson correlation matrix then analyzed using simple linear regression. Analysis and visualization were carried out in SigmaPlot 14 (Systat Software Inc.). For all statistical analyses, alpha was set at 0.05 and both significant (p<0.05) and marginal (0.05<p<0.1) differences were reported.

## 3 Results

**Figure 4:**
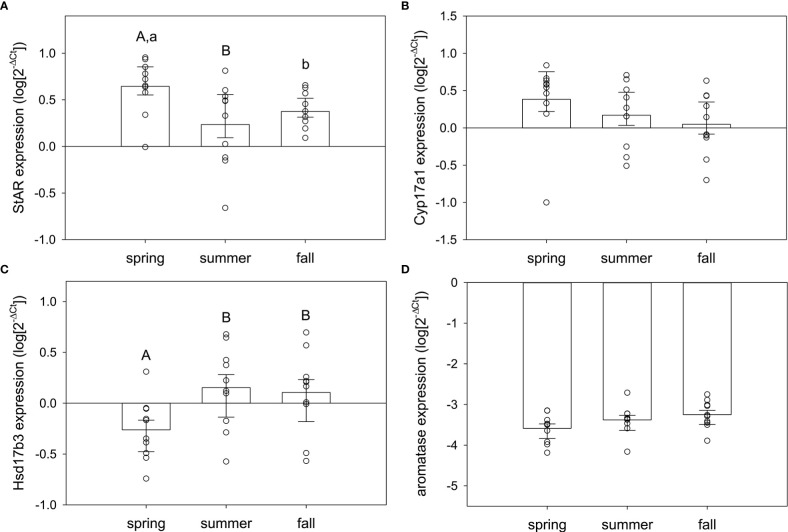
Normalized steroidogenic gene expression in testes from male red-sided garter snakes across three seasons (spring, summer, and fall). Genes: **(A)** StAR, **(B)** Cyp17a1, **(C)** Hsd17b3, **(D)** aromatase (Cyp19a1). Uppercase letters indicate significant differences (p<0.05; lowercase marginal, 0.05<p<0.1). Bars are means (+S.E.M., -95% C.I.). Sample sizes: spring (n=10), summer (n=10), fall (n=10).

In testis, season significantly influenced expression of some, but not all, of the target genes. StAR (F_2,29 =_ 3.97, p=0.031) and Hsd17b3 (F_2,29 =_ 3.71, p=0.037) were seasonally variable, but Cyp17a1 and aromatase were not (F_2,29 =_ 1.37, p=0.27; F_2,29 =_ 2.40, p=0.11, respectively) (**Figure 4**). For StAR (**Figure 4A**), expression was greatest in spring compared to summer (q=3.92, p=0.026) and marginally higher than fall (q=2.58, p=0.079; fall vs. summer, q=1.34, p=0.35). For Hsd17b3 (**Figure 4C**), expression was greatest in summer compared to spring (q=3.52, p=0.049) and fall compared to spring (q=3.12, p=0.036; summer vs. fall, q=0.40, p=0.77).

**Figure 5:**
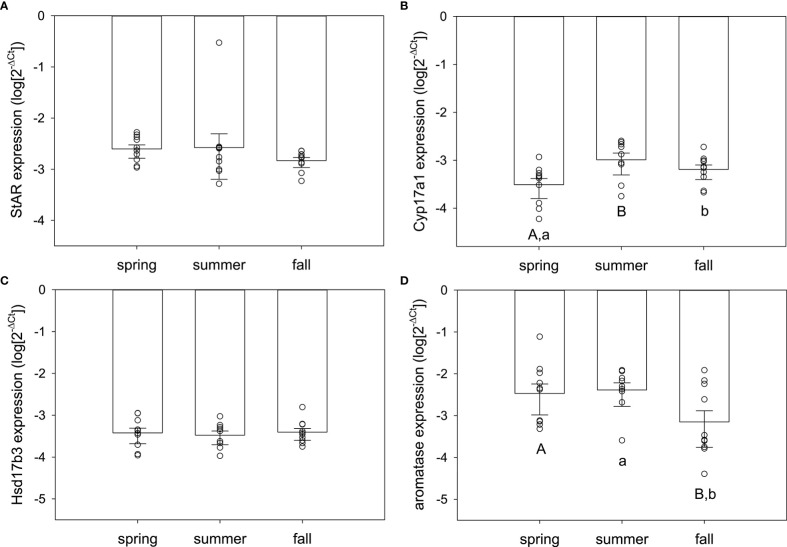
Normalized steroidogenic gene expression in ovarian tissue from female red-sided garter snakes across three seasons (spring, summer, and fall). Genes: **(A)** StAR, **(B)** Cyp17a1, **(C)** Hsd17b3, **(D)** aromatase (Cyp19a1). Uppercase letters indicate significant differences (p<0.05; lowercase marginal, 0.05<p<0.1). Bars are means (+S.E.M., -95% C.I.). Sample sizes: spring (n=10), summer (n=9), fall (n=10).

In ovary, there was significant seasonal variation in Cyp17a1 and aromatase expression but not StAR or Hsd17b3 (**Figure 5**). Cyp17a1 expression was seasonally variable (F_2,28 =_ 4.71, p=0.018), significantly elevated in summer compared to spring (q=4.28, p=0.015), and marginally higher in fall compared to spring (q=2.68, p=0.069) (**Figure 5B**). Summer and fall were not different (q=1.66, p=0.24). Aromatase expression in the ovary was also seasonally variable (F_2,28 =_ 3.38, p=0.049), but there was a different seasonal pattern compared to Cyp17a1 (**Figure 5D**). Aromatase expression was significantly elevated in spring compared to fall (q=3.02, p=0.042) and marginally higher in summer compared to fall (q=3.30, p=0.068). Summer and spring did not differ (q=0.36, p=0.79). No other comparisons were statistically significant.

**Figure 6:**
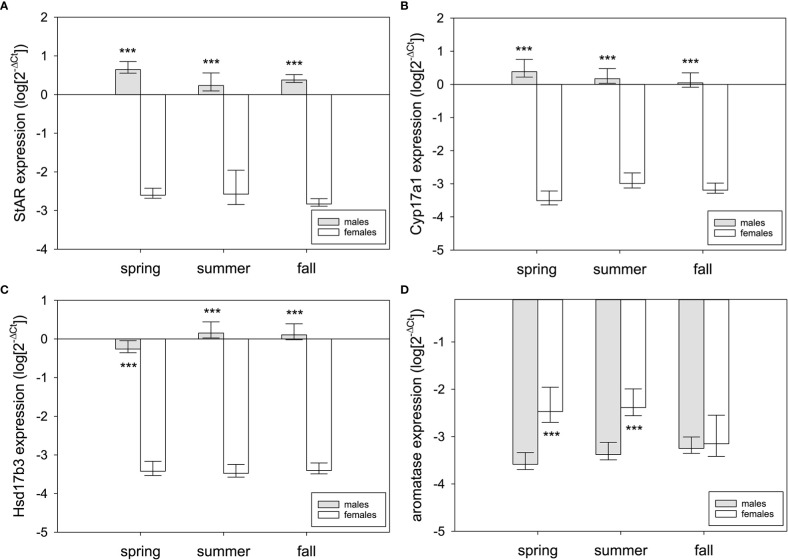
Sex differences in steroidogenic gene expression in the gonads of red-sided garter snakess across three seasons (spring, summer, and fall). Genes: **(A)** StAR, **(B)** Cyp17a1, **(C)** Hsd17b3, **(D)** aromatase (Cyp19a1). Asterisks indicate significant differences (p<0.001). Bars are means (+95% C.I., -S.E.M.). Sample sizes for each bar are n=10 except for summer females (n=9).

Sex differences were tested using two-way ANOVA (sex, season as factors; sex × season interaction). There were extreme sex differences in expression of each steroidogenic enzyme, and those differences were strongly male-biased for all genes but aromatase (**Figure 6**). StAR expression was higher in testis than ovary across all seasons (F_1,58 =_ 824.24, p<0.001; spring: q=24.87, p<0.001; summer: q=20.96, p<0.001; fall: q=24.55, p<0.001) (**Figure 6A**). The same was true for Cyp17a1 (F_1,58 =_ 991.83, p<0.001; spring: q=29.44, p<0.001; summer: q=23.26, p<0.001; fall: q=24.51, p<0.001) and Hsd17b3 (F_1,58 =_ 1,462.48, p<0.001; spring: q=29.01, p<0.001; summer: q=32.42, p<0.001; fall: q=32.21, p<0.001). Aromatase expression higher in ovary than testis (F_1,58 =_ 25.62, p<0.001), but this was only in spring (q=6.33, p<0.001) and summer (q=5.47, p<0.001) (fall: q=0.55, p=0.69) (**Figure 6D**).

**Figure 7:**
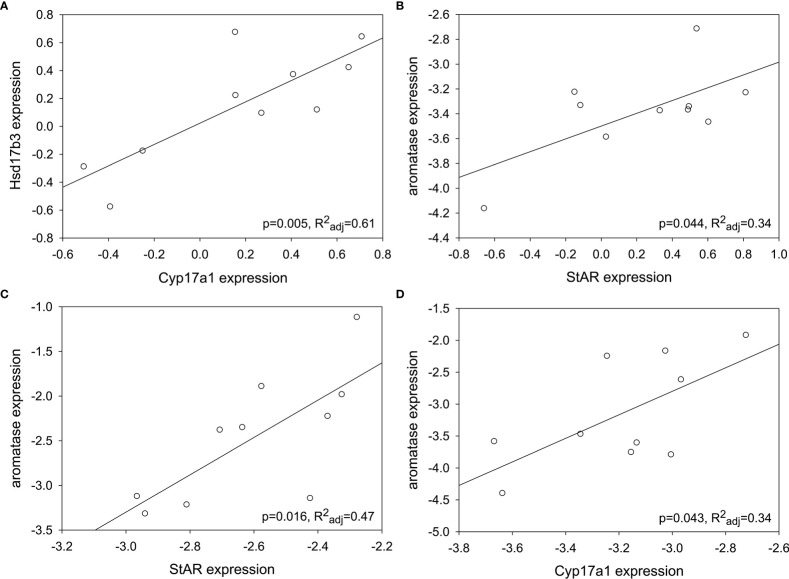
Some steroidogenic genes showed positive correlations in expression depending on gonad type and season. **(A)** Cyp17a1 and Hsd17b3; summer testis. **(B)** StAR and aromatase; summer testis. **(C)** StAR and aromatase; spring ovary. **(D)** Cyp17a1 and aromatase; fall ovary. Target gene expression was normalized (log_10_[2^-ΔCt^]).

Because the target genes in this study form a functional cluster of enzymes in steroidogenesis, Pearson correlation tables were generated per sex per season to determine any associations (**Table S1**). For males, there were positive correlations between genes only in summer: Cyp17a1 and Hsd17b3 (F_1,9 =_ 15.20, p=0.005, R^2^_adj_=0.61; **Figure 7A**) and StAR and aromatase (F_1,9 =_ 5.73, p=0.044, R^2^_adj_=0.34; **Figure 7B**). For females, there were positive correlations between genes in both spring and fall. StAR and aromatase expression were positively correlated in spring (F_1,9 =_ 9.24, p=0.016, R^2^_adj_=0.47; **Figure 7C**), and Cyp17a1 and aromatase expression were positively correlated in fall (F_1,9 =_ 5.75, p=0.043, R^2^_adj_=0.34; **Figure 7D**). There was a marginal positive correlation between StAR and Cyp17a1 in spring (F_1,9 =_ 4.07, p=0.078, R^2^_adj_=0.25) and Cyp17a1 and Hsd17b3 in fall (F_1,9 =_ 3.72, p=0.09, R^2^_adj_=0.23). No other correlations in expression between genes were detected in testis or ovary.

## 4 Discussion

Contrary to expectations based on the timing of spermatogenesis and the known peak in circulating testosterone for male red-sided garter snakes, StAR was maximally expressed in the testes during the spring mating season. In vertebrates, StAR is widely purported as the rate-limiting enzyme in the steroidogenic pathway (47–50). The next enzyme analyzed in the steroidogenic pathway, Cyp17a1, also showed highest expression in spring testis, though this was not statistically significant. These results match what would be predicted for a species with an associated reproductive pattern: our observations suggest active investment in steroidogenesis when mating behavior is maximal in male *Thamnophis sirtalis parietalis*. However, it is paradoxical in the context of a species exhibiting a dissociated reproductive strategy. Male red-sided garter snakes do not undergo spermatogenesis during the spring breeding season, nor do they have significantly elevated testosterone relative to the rest of the active season. Moreover, male courtship behavior in this species is not induced by elevated testosterone (18); rather, it is winter dormancy that alters neuronal steroidogenesis to upregulate sexually dimorphic regions of the hypothalamus and activate sex behavior (42). Peak spermatogenesis and increased circulating testosterone will not occur until late summer, thus the enzymes in this pathway should be upregulated in sync with or tightly coupled to these events (15, 51). Our data only partially support this: Hsd17b3 was elevated in summer and remained high into fall. Though StAR expression was highest in spring, expression of StAR in summer was strongly, positively correlated with the peak of Hsd17b3 expression, indicating that steroidogenesis in summer is maximizing testosterone production in sync with gametogenesis as predicted by previous research (9, 15, 52). Lastly, we also observed greatest individual variation in Hsd17b3 expression compared to the other steroidogenic genes, which suggests this gene may be a source of phenotypic variation in male red-sided garter snakes.

At emergence from low-temperature dormancy in spring, the testes of male *Thamnophis sirtalis parietalis* are regressed, and thus no major steroidogenic activity was predicted. However, our results strongly suggest that steroidogenesis is occurring–and much earlier than expected. Perhaps males are front-loading precursor androgens in anticipation of summer gametogenesis. Circulating testosterone levels can be elevated in male garter snakes upon emergence from hibernation, but as soon as they actively begin courting females at the den these residual androgens are cleared from circulation (15, 16, 53). The testis may be able to store weaker androgens (e.g., DHEA, androstenedione) as substrates for immediate conversion to more potent androgens (e.g., testosterone) once downstream enzymes are upregulated/active. Thus, steps in androgenesis may occur seasonally in male red-sided garter snakes. Tissue-specific measurement of sex steroids would illuminate this idea, as has been demonstrated recently in brown anoles (*Anolis sagrei*) (39). Our results also beg the question of how environmental stimuli may act differentially on specific enzymes within the same tissue, analogous to temperature-dependent allozymes in cold-adapted fish species (54). Suites of steroidogenic genes may be tuned to specific temperature optima in red-sided garter snakes to result in distinctly seasonal, step-wise regulation of steroidogenic enzymes. Future research in our laboratory will address this possibility.

Female red-sided garter snakes appear to be limited in the synthesis of estrogens based on our results, with maximal expression of the terminal enzyme, aromatase, occurring during the predicted peak in estrogen synthesis (spring). The rate-limiting enzyme in steroidogenesis, StAR, was maximally expressed in spring and was positively correlated with aromatase expression, suggesting estrogen biosynthesis is most active during the mating season. Females produce a mating-induced E_2_ surge in spring that is aromatase-dependent (23). Further, the E_2_ surge can be induced in *ex vivo* preparations of ovarian fragments from female garter snakes in spring *via* prostaglandin treatment (55, 56). Seasonal, anticipatory upregulation of ovarian aromatase would be beneficial in maximizing E_2_ synthesis given the important roles of estrogen signaling in female reproductive physiology. However, the broadscale reduction in aromatase expression we observed is unusual. The females in our study were unmated; how and to what extent mating in spring may shift gene expression in the ovary is an outstanding question. Perhaps we have observed facultative quiescence in steroidogenic gene expression, especially as there are many examples of social behavior inducing changes in gene expression at each level of the vertebrate hypothalamic-pituitary-gonadal axis (reviewed in 56). By extension, female garter snakes may thus have significant phenotypic plasticity in steroidogenesis that is dependent upon spring mating. As with the testes, ovarian Cyp17a1 was upregulated in summer compared to spring. This did not match our expectation that StAR would be the major enzyme with seasonal variation. Previous work in this species indicated that steroidogenesis was mating dependent: E_2_ peaks immediately but briefly after mating (approximately 6 hours post-copulation; returns to baseline by 24 hours) and then remains relatively low for the remainder of the year (23, 55). Through the examination of upstream mechanisms (steroidogenic enzymes), we have partially explained one limitation for female production of estrogens in red-sided garter snakes.

The seasonal pattern of steroidogenesis in females is likely tied to the unresolved mechanisms regulating vitellogenesis in garter snakes. Vitellogenesis is crucial for gestation and parturition of offspring because vitellogenins serve as lipid transport proteins and egg-yolk precursors that facilitate lipid circulation for follicular development by the ovaries (24, 57). The exact mechanisms that control and induce vitellogenesis in garter snakes are unclear, as unmated females in a given year can become vitellogenic without experiencing the mating-induced E_2_ surge (23). Instead, it was hypothesized that these females mated the previous fall and experienced a post-mating E_2_ surge, initiated then halted vitellogenesis prior to winter dormancy, and eventually resumed vitellogenesis the next spring (20, 23). Further, in the ovarian *ex vivo* experiment mentioned above, E_2_ synthesis was significantly variable: the ovaries of some females were indicated as “unresponsive” due to no change in E_2_ secretion following prostaglandin treatment (56). Other studies have shown that body condition at emergence may also predict vitellogenesis; however, this was not consistent as some females in poor condition still became vitellogenic (58). These separate studies help explain the general variation in steroidogenic gene expression we observed between individual females for both Cyp17a1 and aromatase, and this variation was not as pronounced for StAR or Hsd17b3.

Female pheromone production in garter snakes is directly associated with estrogen availability. Across the spring breeding season, females become lose their attractivity because of a reduction in pheromone quality and quantity associated with declining circulating estradiol (59). If ovariectomized in spring, females have reduced attractivity the next spring (21). Systemic aromatase inhibition across the active year (spring to fall) reduces female pheromone production and attractivity the next spring (26). Further, male garter snakes given estradiol implants produce abundant female sex pheromone and are attractive to wild males, an effect lost once the implants are removed which indicates an activational role for estradiol (25). However, the seasonal relationship between ovarian status, circulating estradiol, and female pheromone production is still unclear. Female garter snakes ovariectomized in fall or during hibernation remain attractive the next spring (60), mating renders females unattractive despite the reflexive surge in estradiol (20, 23), and low temperature dormancy increases pheromone quality and quantity (61). Female garter snakes may thus have constitutive production of sex pheromone from a skin that is organized at some point during early life to be relatively independent of circulating estradiol (25).

Our study is confined by our focus on mRNA expression, which does not always correspond with protein production or activity. For example, at least one previous study indicated a mismatch between mRNA expression and gene activity for steroidogenic genes (38). Our analyses only utilized cDNA, but protein quantification and functional assays should also be pursued to determine the outcomes of the observed seasonal and sexual patterns of mRNA expression. Measurements of steroidogenic conversion of specific hormones in gonadal and extra-gonadal tissues would be particularly informative. We observed substantial variation in gene expression among individuals, especially females; as stated above, that variation is biological and informative. The gonadotropins (LH, FSH) strongly influence the rate of gonadal steroidogenesis (e.g., 62), but molecular identification and purification of gonadotropins in squamates (snakes, lizards) has been challenging and, to our knowledge, remains elusive in snakes (e.g., 63, 64). The gene for FSHβ is annotated for *Thamnophis sirtalis* (NCBI reference sequence NW_013658033.1), and recent work has demonstrated a key role for GnRH in neural plasticity of garter snakes (65); thus, additional questions could be pursued across the hypothalamic-pituitary-gonadal axis to understand the relative influence of tropic hormones on steroidogenesis. Lastly, we did not focus on quantifying steroid hormone levels in our study because the patterns of testosterone and estradiol production are well-established in *Thamnophis sirtalis parietalis*; however, a survey of additional sex steroids, especially DHEA, is warranted to support and extend our conclusions.

Our data suggest that the reproductive pattern of red-sided garter snakes, as is the case for many other snake species, is a mix of dissociation and association between steroidogenesis and mating activity. Many pitviper species show mixed reproductive patterns, with spermatogenesis occurring in late summer but breeding seasons varying per species and even population (10, 66). Eastern copperheads (*Agkistrodon contortrix*) and pygmy rattlesnakes (S*istrurus miliarius*) have bimodal breeding seasons but only unimodal gametogenesis; thus, these species exhibit both associated and dissociated reproductive patterns in a single annual cycle (67, 68). Contrastingly, European asps (*Vipera aspis*) and western diamondback rattlesnakes (*Crotalus atrox*) have unimodal increases in circulating sex steroids uncoupled from their unimodal breeding seasons (69, 70). Lastly, the type of reproductive pattern can even be sex-specific, as with the cottonmouth (*Agkistrodon piscivorus*) (66). Our results further call into question the associated-dissociated binary when describing hormone-behavior patterns in vertebrate reproduction, as others have suggested (71). Such labels are restrictive, and it may be more pertinent to treat reproductive patterns as a continuum rather than a binary.

Our study shows that steroidogenic enzyme expression in the gonads is seasonally and sexually variable in red-sided garter snakes. A notable question centers on the importance of winter dormancy in the regulation of the steroidogenic pathway. The growing body of evidence suggests that beyond the central control of male courtship behavior, prolonged low-temperature dormancy is a critical period of significant physiological shifts in the annual cycle of red-sided garter snakes (e.g., 44, 61, 65, 72). How temperature is influencing gene expression in the gonads also holds implications for climate change—namely how reduced periods of dormancy and subsequent, diffuse spring breeding seasons may relax selection on steroidogenesis in this temperature-dependent system.

## Data availability statement

The original contributions presented in the study are included in the article/**Supplementary Material** further inquiries can be directed to the corresponding author/s.

## Ethics statement

The animal study was reviewed and approved by JMU Institutional Animal Care and Use Committee.

## Author contributions

JL, MB, and MP conceived of and designed the study. JL and MB conducted all of the laboratory work and collected and organized all data. HR maintained the animals and collected tissues. MP performed statistical analyses. MB and JL wrote the first draft of the manuscript. All authors contributed to manuscript revision, read, and approved the submitted version.

## References

[1] Nelson RJ Badura LL Goldman BD. Mechanisms of seasonal cycles of behavior. Ann Rev Psychol (1990) 41:81–108. doi: 10.1146/annurev.ps.41.020190.000501 2407180

[2] Omura T Morohashi K. Gene regulation of steroidogenesis. J Steroid Biochem Molec Biol (1995) 53(1):19–25. doi: 10.1016/0960-0760(95)00036-Y 7626452

[3] Schulz RW de França LR Lareyre JJ LeGac F Chiarini-Garcia H Nobrega RH. Spermatogenesis in fish. Gen Comp Endocr (2010) 165(3):390–411. doi: 10.1016/j.ygcen.2009.02.013 19348807

[4] Ho S-M Kleis S McPherson R Heisermann GJ Callard IP. Regulation of vitellogenesis in reptiles. Herpetologica (1982) 38(1):40–50.

[5] Adkins-Regan E. Hormonal organization and activation: evolutionary implications and questions. Gen Comp Endocr (2012) 176:279–85. doi: 10.1016/j.ygcen.2011.12.040 22248442

[6] Ball GF Balthazart J. Neuroendocrine regulation of reproductive behavior in birds. In: Pfaff DW Arnold AP Etgen AM Fahrbach SE Rubin RT, editors. Hormones, brain and behavior. (2009). Elsevier Academic Press. p. 855–95.

[7] Wilczynski W Lynch KS. Female sexual arousal in amphibians. Horm Behav (2011) 59(5):630–6. doi: 10.1016/j.yhbeh.2010.08.015 PMC3008597 20816968

[8] Itoh M Ishuii S. Changes in plasma levels of gonadotropins and sex steroid in the toad, *Bufo japonicus*, in association with behavior during the breeding season. Gen Comp Endocrinol (1990) 80(3):451–64. doi: 10.1016/0016-6480(90)90194-Q 2127034

[9] Crews D. Gamete production, sex hormone secretion, and mating behavior uncoupled. Horm Behav (1984) 18(1):22–8. doi: 10.1016/0018-506X(84)90047-3 6538547

[10] Taylor EN DeNardo DF. Hormones and reproductive cycles in snakes. In: Norris DO Lopez KH, editors. Hormones and reproduction of vertebrates: Reptiles. London, UK: Academic Press. (2001) p. 355–72.

[11] Crews D Moore MC. Evolution of mechanisms controlling mating behavior. Science (1986) 231(4734):121–5. doi: 10.1126/science.3941893 3941893

[12] Woolley SC Sakata JT Crews D. Evolutionary insights into the regulation of courtship behavior in male amphibians and reptiles. Physiol Behav (2004) 83(2):347–60. doi: 10.1016/j.physbeh.2004.08.021 15488550

[13] Shine R Brown GP. Adapting to the unpredictable: reproductive biology of vertebrates in the Australian wet-dry tropics. Phil Trans R Soc B (2008) 363(1490):363–73. doi: 10.1098/rstb.2007.2144 PMC2606755 17638689

[14] Krohmer RW Grassman M Crews D. Annual reproductive cycle in the male red-sided garter snake, *Thamnophis sirtalis parietalis*: field and laboratory studies. Gen Comp Endocr (1987) 68:64–75. doi: 10.1016/0016-6480(87)90061-X 3666424

[15] Krohmer RW. The male red-sided garter snake (*Thamnophis sirtalis parietalis*): reproductive pattern and behavior. ILAR J (2004) 45(1):65–74. doi: 10.1093/ilar.45.1.65 14756156

[16] Lutterschmidt DI. Chronobiology of reproduction in garter snakes: neuroendocrine mechanisms and geographic variation. Gen Comp Endocr (2012) 176(3):448–55. doi: 10.1016/j.ygcen.2011.12.015 22210163

[17] Garstka WR Camazine B Crews D. Interactions of behavior and physiology during the annual reproductive cycle of the red-sided garter snake (*Thamnophis sirtalis parietalis*). Herpetologica (1982) 38(1):104–23.

[18] Crews D Camazine B Diamond M Mason R Tokarz RR Garstka WR. Hormonal independence of courtship behavior in the male garter snake. Horm Behav (1984) 18(1):29–41. doi: 10.1016/0018-506X(84)90048-5 6706317

[19] O’Donnell RP Shine R Mason RT. Seasonal anorexia in the male red-sided garter snake, thamnophis sirtalis parietalis. Behav Ecol Sociobiol (2004) 56:413–9. doi: 10.1007/s00265-004-0801-x

[20] Mendonça MT Crews D. Effect of fall mating on ovarian development in the red-sided garter snake. Am J Physiol - Reg I (1989) 257(6):R1548–50. doi: 10.1152/ajpregu.1989.257.6.R1548 2604010

[21] Mendonça MT Crews D. Control of attractivity and receptivity in female red-sided garter snakes. Horm Behav (2001) 40(1):43–50. doi: 10.1006/hbeh.2001.1665 11467883

[22] Whittier JM Crews D. Ovarian development in red-sided garter snakes, *Thamnophis sirtalis parietalis*: relationship to mating. Gen Comp Endocr (1986) 61(1):5–12. doi: 10.1016/0016-6480(86)90243-1 3940930

[23] Whittier JM Mason RT Crews D. Plasma steroid hormone levels of female red-sided garter snakes, *Thamnophis sirtalis parietalis*: relationship to mating and gestation. Gen Comp Endocr (1987) 67:33–43. doi: 10.1016/0016-6480(87)90202-4 3623067

[24] Garstka GR Tokarz RR Diamond M Halpert A Crews D. Behavioral and physiological control of yolk synthesis and deposition in the female red-sided garter snake (*Thamnophis sirtalis parietalis*). Horm Behav (1985) 9(2):137–53. doi: 10.1016/0018-506X(85)90014-5 3924812

[25] Parker MR Mason RT. How to make a sexy snake: estrogen activation of female sex pheromone in male red-sided garter snakes. J Exp Biol (2012) 215:723–30. doi: 10.1242/jeb.064923 22323194

[26] Rucker HR Parker MR. Decreased attractivity in female garter snakes treated with an aromatase inhibitor. J Exp Zool Part A (2022) 337(2):171–80. doi: 10.1002/jez.2546 34533896

[27] Norris D Carr J. Vertebrate endocrinology. United States: Academic Press (2020).

[28] Lutterschmidt DI LeMaster MP Mason RT. Minimal overwintering temperatures of red-sided garter snakes (*Thamnophis sirtalis parietalis*): a possible cue for emergence? Can J Zool (2006) 84:771–7. doi: 10.1139/z06-043

[29] Shine R LeMaster MP Moore IT Olsson MM Mason RT. Bumpus in the snake den: effects of sex, size, and body condition on mortality of red-sided garter snakes. Evolution (2001) 55(3):598–604. doi: 10.1554/0014-3820(2001)055[0598:BITSDE]2.0.CO;2 11327166

[30] Canale CI Henry PY. Adaptive phenotypic plasticity and resilience of vertebrates to increasing climatic unpredictability. Clim Res (2010) 43:135–47. doi: 10.3354/cr00897

[31] Leduc M Mailhot A Frigon A Martel JL Ludwig R Brietzke GB. The ClimEx project: A 50-member ensemble of climate change projections at 12-km resolution over Europe and northeastern north America with the Canadian regional climate model (CRCM5). J Appl Meteorol Climatol (2019) 58(4):663–93. doi: 10.1175/JAMC-D-18-0021.1

[32] Little AG Seebacher F. Acclimation, acclimatization, and seasonal variation in amphibians and reptiles. In: Vieira de Andrade D Bevier CV de Carvalho JE, editors. Amphibian and reptile adaptations to the environment. Boca Raton: CRC Press (2016). p. 41–55.

[33] Rajakumar A Senthilkumaran B. Expression analysis of cyp11a1 during gonadal development, recrudescence and after hCG induction and sex steroid analog treatment in the catfish, *Clarias batrachus* . Comp Biochem Physiol B (2014) 176:42–7. doi: 10.1016/j.cbpb.2014.07.007 25107325

[34] Canosa LF Ceballos NR. Seasonal changes in testicular steroidogenesis in the toad *Bufo arenarum* h. Gen Comp Endocr (2002) 125(3):426–34. doi: 10.1006/gcen.2001.7768 11884086

[35] Peek CE Cohen RE. Seasonal regulation of steroidogenic enzyme expression within the green anole lizard (*Anolis carolinensis*) brain and gonad. Gen Comp Endocr (2018) 268:88–95. doi: 10.1016/j.ygcen.2018.08.005 30077794

[36] Rosati L Di Fiore MM Prisco M Di Giacomo Russo F Venditti M Andreuccetti P. Seasonal expression and cellular distribution of star and steroidogenic enzymes in quail testis. J Exp Zool B (2019) 332(6):198–209. doi: 10.1002/jez.b.22896 31433565

[37] Mukherjee A Haldar C. Photoperiodic regulation of melatonin membrane receptor (MT1R) expression and steroidogenesis in testis of adult golden hamster, mesocricetus auratus. J Photochem Photobiol B (2014) 140:374–80. doi: 10.1016/j.jphotobiol.2014.08.022 25255424

[38] Rocha A Zanuy S Carrillo M Gomez A. Seasonal changes in gonadal expression of gonadotropin receptors, steroidogenic acute regulatory protein and steroidogenic enzymes in the European sea bass. Gen Comp Endocr (2009) 162(3):265–75. doi: 10.1016/j.ygcen.2009.03.023 19345689

[39] Himmelstein R Spahija A Fokidis HB. Evidence for fasting induced extra-adrenal steroidogenesis in the male brown anole, *Anolis sagrei* . Comp Biochem Physiol B (2021) 253:110544. doi: 10.1016/j.cbpb.2020.110544 33338607

[40] Lema SC. Hormones and phenotypic plasticity in an ecological context: linking physiological mechanisms to evolutionary processes. Integr Comp Biol (2014) 54(5):850–63. doi: 10.1093/icb/icu019 24752548

[41] Cruze L Kohno S McCoy MW Guillette LJ Jr. Towards an understanding of the evolution of the chorioallantoic placenta: steroid biosynthesis and steroid hormone signaling in the chorioallantoic membrane of an oviparous reptile. Biol Reprod (2012) 87(3):1–11. doi: 10.1095/biolreprod.112.101360 22811568

[42] Krohmer RW Boyle MH Lutterschmidt DI Mason RT. Seasonal aromatase activity in the brain of the male red-sided garter snake. Horm Behav (2010) 58(3):485–92. doi: 10.1016/j.yhbeh.2010.04.011 20420841

[43] Lutterschmidt DI Mason RT. Geographic variation in timekeeping systems among three populations of garter snakes (*Thamnophis sirtalis*) in a common garden. Physiol Biochem Zool (2008) 81(6):810–25. doi: 10.1086/589900 18937566

[44] Lutterschmidt DI Mason RT. Endocrine mechanisms mediating temperature-induced reproductive behavior in red-sided garter snakes (*Thamnophis sirtalis parietalis*). J Exp Biol (2009) 212(19):3108–18. doi: 10.1242/jeb.033100 19749103

[45] Ashton SE Vernasco BJ Moore IT Parker MR. Sex and seasonal differences in mRNA expression of estrogen receptor α (ESR1) in red-sided garter snakes (*Thamnophis sirtalis parietalis*). Gen Comp Endocr (2018) 267:59–65. doi: 10.1016/j.ygcen.2018.05.026 29807033

[46] Bona-Gallo A Licht P. Effects of temperature on sexual receptivity and ovarian recrudescence in the garter snake, thamnophis sirtalis parietalis. Herpetologica (1983) 39(2):173–82.

[47] Stocco DM Clark BJ. Role of the steroidogenic acute regulatory protein (StAR) in steroidogenesis. Biochem Pharmacol (1996) 51(3):197–205. doi: 10.1016/0006-2952(95)02093-4 8573184

[48] Stocco DM. Recent advances in the role of StAR. Rev Reprod (1998) 3:82–5. doi: 10.1530/ror.0.0030082 9685186

[49] Hanukoglu I Hanukoglu Z. Stoichiometry of mitochondrial cytochromes p-450, adrenodoxin and adrenodoxin reductase in adrenal cortex and corpus luteum. Eur J Biochem (1986) 157:27–31. doi: 10.1111/j.1432-1033.1986.tb09633.x 3011431

[50] Clark BJ Stocco DM. StAR–a tissue specific acute mediator of steroidogenesis. Trends Endocrin Met (1996) 7(7):227–33. doi: 10.1016/S1043-2760(96)00114-2 18406752

[51] Krohmer RW Jurkovic J. Neuronal plasticity in the forebrain of the male red-sided garter snake: Effect of season, low temperature dormancy, and hormonal status on dendritic spine density. Physiol Behav (2020) 215(1):112789. doi: 10.1016/j.physbeh.2019.112789 31866231

[52] Crews D. Trans-seasonal action of androgen in the control of spring courtship behavior in male red-sided garter snakes. Proc Natl Acad Sci USA (1991) 88(9):3545–8. doi: 10.1073/pnas.88.9.3545 PMC51488 2023900

[53] Cease AJ Lutterschmidt DI Mason RT. Corticosterone and the transition from courtship behavior to dispersal in male red-sided garter snakes (*Thamnophis sirtalis parietalis*). Gen Comp Endocr (2007) 150(1):124–31. doi: 10.1016/j.ygcen.2006.07.022 16989831

[54] Hardewig I van Dijk PLM Moyes CD Pörtner HO. Temperature-dependent expression of cytochrome-c oxidase in Antarctic and temperate fish. Am J Physiol - Reg I (1999) 277(2):R508–16. doi: 10.1152/ajpregu.1999.277.2.R508 10444558

[55] Whittier JM Crews D. Mating increases plasma levels of prostaglandin F2α in female garter snakes. Prostaglandin (1986) 37(3):359–66. doi: 10.1016/0090-6980(89)90006-3 2748917

[56] Whittier JM O’Conner CS. Prostaglandin F_2α_, sexual behavior and ovarian estrogen synthesis in garter snakes (*Thamnophis sirtalis parietalis*). Comp Biochem Physiol (1991) 100A(4):881–5. doi: 10.1016/0300-9629(91)90308-Y 1685380

[57] Babin PJ Bogerd J Kooiman FP Van Marrewijk WJ van der Horst DJ. Apolipophorin II/I, apolipoprotein b, vitellogenin, and microsomal triglyceride transfer protein genes are derived from a common ancestor. J Mol Evol (1999) 49(1):150–60. doi: 10.1007/PL00006528 10368443

[58] Whittier JM Crews D. Body mass and reproduction in female red-sided garter snakes (*Thamnophis sirtalis parietalis*). Herpetologica (1990) 46(2):219–26.

[59] Uhrig EJ Lutterschmidt DI Mason RT LeMaster MP. Pheromonal mediation of intraseasonal declines in the attractivity of female red-sided garter snakes, thamnophis sirtalis parietalis. J Chem Ecol (2012) 38:71–80. doi: 10.1007/s10886-011-0054-x 22231473

[60] Mendonça MT Crews D. Effects of ovariectomy and estrogen replacement on attractivity and receptivity in the red-sided garter snake (*Thamnophis sirtalis parietalis*). J Comp Physiol A (1996) 178:373–81. doi: 10.1007/BF00193975 8583424

[61] Parker MR Mason RT. Low temperature dormancy affects the quantity and quality of the female sexual attractiveness pheromone in red-sided garter snakes. J Chem Ecol (2009) 35(10):1234–41. doi: 10.1007/s10886-009-9699-0 19904571

[62] Tripathy M Rai U. Temporal expression and gonadotropic regulation of aromatase and estrogen receptors in the ovary of wall lizard, *Hemidactylus flaviviridis*: correlation with plasma estradiol and ovarian follicular development. Steroids (2017) 128:23–31. doi: 10.1016/j.steroids.2017.10.005 29042199

[63] Licht P Farmer SW Bona-Gallo A Papkoff H. Pituitary gonadotropins in snakes. Gen Comp Endocr (1979) 39:34–52. doi: 10.1016/0016-6480(79)90190-4 488693

[64] Bluhm AP Toledo RA Mesquita FM Pimenta MT Fernandes FM Ribela MT. Molecular cloning, sequence analysis and expression of the snake follicle-stimulating hormone receptor. Gen Comp Endocr (2004) 137(3):300–11. doi: 10.1016/j.ygcen.2004.03.014 15201068

[65] Lutterschmidt DI Lucas AR Summers AR. Trans-seasonal activation of the neuroendocrine reproductive axis: Low-temperature winter dormancy modulates gonadotropin-releasing hormone neurons in garter snakes. J Exp Zool Part A (2022) 337(1):50–64. doi: 10.1002/jez.2506 34270177

[66] Siegel DS Sever DM Rheubert JL Gribbins KM. Reproductive biology of *Agkistrodon piscivorus* lacépède (Squamata, serpentes, viperidae, crotalinae). Herpetol Monogr (2009) 23:74–107. doi: 10.1655/08-031.1

[67] Maruska KP Fernald RD. Social regulation of gene expression in the hypothalamic-pituitary-gonadal axis. Physiology (2011) 26(6):412–23. doi: 10.1152/physiol.00032.2011 22170959

[68] Lind CM Moore IT Vernasco BJ Latney LV DiGeronimo PM Farrell TM. The relationship between steroid hormone and seasonal reproductive events in free-living female pygmy rattlesnakes, sistrurus miliarius. Gen Comp Endocr (2020) 290:113416. doi: 10.1016/j.ygcen.2020.113416 32006531

[69] Saint Girons H Bradshaw SD Bradshaw FJ. Sexual activity and plasma levels of sex steroids in the aspic viper *Vipera aspis* l. (Reptilia, viperidae). Gen Comp Endocr (1993) 91(3):287–97. doi: 10.1006/gcen.1993.1129 8224772

[70] Taylor EN DeNardo DF Jennings DH. Seasonal steroid hormone levels and their relation to reproduction in the Western diamond-backed rattlesnake, *Crotalus atrox* (Serpentes: Viperidae). Gen Comp Endocr (2004) 136(3):328–37. doi: 10.1016/j.ygcen.2004.01.008 15081832

[71] Benner SL Woodley SK. The reproductive pattern of male dusky salamanders (genus *Desmognathus*) is neither associated nor dissociated. Horm Behav (2007) 51(4):542–7. doi: 10.1016/j.yhbeh.2007.02.004 17382330

[72] Hubert DL. Life in a rapidly changing environment: the thermal biology of the red-sided garter snake (Thamnophis sirtalis parietalis). Corvallis, (OR: Oregon State University (2002).

