# Supplementary figures and images for: Reconsidering reproductive patterns in a model dissociated species, the red-sided garter snake: Sex-specific and seasonal changes in gonadal steroidogenic gene expression
Source: Front Endocrinol (Lausanne). 2023 Mar 13;14:1135535. doi: 10.3389/fendo.2023.1135535 (PMC10040831; doi:10.3389/fendo.2023.1135535)

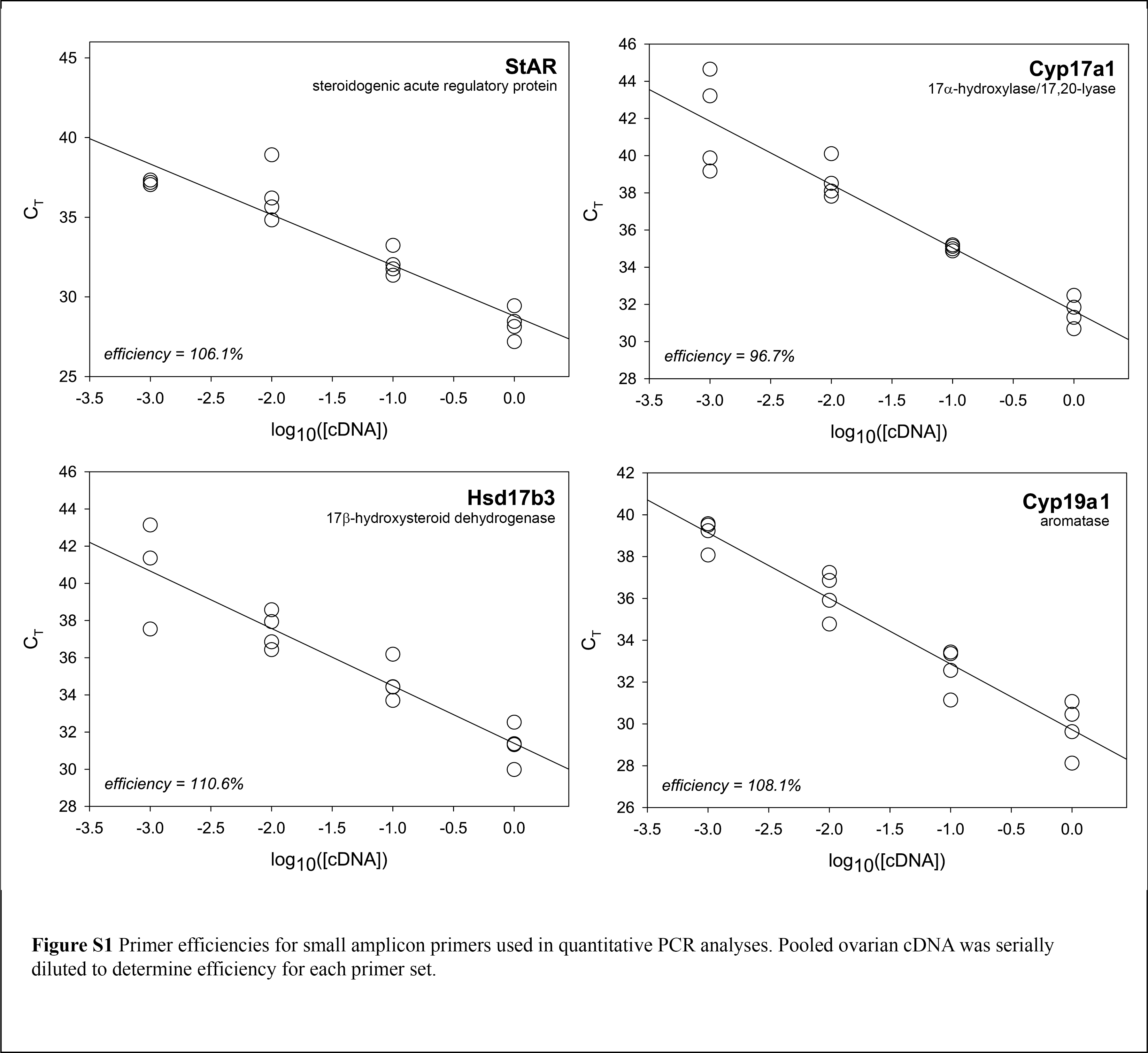

Supplement: Supplementary file 1 [file Image_1.jpeg]
