# Supplementary material for: Reconsidering reproductive patterns in a model dissociated species, the red-sided garter snake: Sex-specific and seasonal changes in gonadal steroidogenic gene expression
Source: Front Endocrinol (Lausanne). 2023 Mar 13;14:1135535. doi: 10.3389/fendo.2023.1135535 (PMC10040831; doi:10.3389/fendo.2023.1135535)
Supplement: Supplementary file 2 [file Table_1.docx]

**Table S1** Pearson correlation values for pairs of target genes based on relative expression per sex per season (correlation coefficient; p-value). Significant values (p<0.05) are in bold, marginal values (0.05<p<0.1) are underlined.

| spring |  |  |  |  |  |  | |  | |  | |
| --- | --- | --- | --- | --- | --- | --- | --- | --- | --- | --- | --- |
| males |  |  |  |  | females |  | |  | |  | |
| gene | Cyp17a1 | Hsd17b3 | aromatase |  |  | | Cyp17a1 | | Hsd17b3 | | aromatase |
| StAR | 0.54; 0.10 | -0.54; 0.10 | 0.44; 0.19 |  | StAR | | 0.58; 0.07 | | 0.31; 0.37 | | 0.73; **0.01** |
| Cyp17a1 | -- | 0.10; 0.78 | -0.23; 0.51 |  | Cyp17a1 | | -- | | -0.24; 0.49 | | 0.26; 0.46 |
| Hsd17b3 | -- | -- | -0.23; 0.51 |  | Hsd17b3 | | -- | | -- | | 0.25; 0.47 |
|  |  |  |  |  |  |  | |  | |  | |
| summer |  |  |  |  |  | |  | |  | |  |
| males |  |  |  |  | females | |  | |  | |  |
|  | Cyp17a1 | Hsd17b3 | aromatase |  |  | | Cyp17a1 | | Hsd17b3 | | aromatase |
| StAR | 0.41; 0.23 | 0.02; 0.94 | 0.64; **0.04** |  | StAR | | 0.44; 0.22 | | 0.18; 0.62 | | 0.38; 0.30 |
| Cyp17a1 | -- | 0.80; **0.004** | 0.44; 0.19 |  | Cyp17a1 | | -- | | 0.08; 0.82 | | 0.51; 0.15 |
| Hsd17b3 | -- | -- | 0.17; 0.62 |  | Hsd17b3 | | -- | | -- | | 0.09; 0.81 |
|  |  |  |  |  |  | |  | |  | |  |
| fall |  |  |  |  |  | |  | |  | |  |
| males |  |  |  |  | females | |  | |  | |  |
|  | Cyp17a1 | Hsd17b3 | aromatase |  |  | | Cyp17a1 | | Hsd17b3 | | aromatase |
| StAR | 0.20; 0.57 | -0.26; 0.46 | 0.15; 0.67 |  | StAR | | 0.53; 0.10 | | -0.41; 0.23 | | 0.47; 0.16 |
| Cyp17a1 | -- | 0.18; 0.60 | 0.17; 0.62 |  | Cyp17a1 | | -- | | -0.56; 0.08 | | 0.64; **0.04** |
| Hsd17b3 | -- | -- | 0.19; 0.58 |  | Hsd17b3 | | -- | | -- | | -0.18; 0.61 |
|  |  |  |  |  |  |  | |  | |  | |
